# Ca^2+^-Permeable AMPA Receptors Contribute to Changed Dorsal Horn Neuronal Firing and Inflammatory Pain

**DOI:** 10.3390/ijms24032341

**Published:** 2023-01-25

**Authors:** Olga Kopach, Yulia Dobropolska, Pavel Belan, Nana Voitenko

**Affiliations:** 1 Bogomoletz Institute of Physiology, 01024 Kyiv, Ukraine; 2 Queen Square Institute of Neurology, University College London, London WC1N 3BG, UK; 3 Department of Biomedicine and Neuroscience, Kyiv Academic University, 03142 Kyiv, Ukraine; 4 Research Center, Dobrobut Academy Medical School, 03022 Kyiv, Ukraine

**Keywords:** chronic pain, persistent peripheral inflammation, dorsal horn neurons, pain signalling, neuronal excitability, action potential firing, molecular targets, AMPA receptors, GluA1-GluA2 subunits, PKCα

## Abstract

The dorsal horn (DH) neurons of the spinal cord play a critical role in nociceptive input integration and processing in the central nervous system. Engaged neuronal classes and cell-specific excitability shape nociceptive computation within the DH. The DH hyperexcitability (central sensitisation) has been considered a fundamental mechanism in mediating nociceptive hypersensitivity, with the proven role of Ca^2+^-permeable AMPA receptors (AMPARs). However, whether and how the DH hyperexcitability relates to changes in action potential (AP) parameters in DH neurons and if Ca^2+^-permeable AMPARs contribute to these changes remain unknown. We examined the cell-class heterogeneity of APs generated by DH neurons in inflammatory pain conditions to address these. Inflammatory-induced peripheral hypersensitivity increased DH neuronal excitability. We found changes in the AP threshold and amplitude but not kinetics (spike waveform) in DH neurons generating sustained or initial bursts of firing patterns. In contrast, there were no changes in AP parameters in the DH neurons displaying a single spike firing pattern. Genetic knockdown of the molecular mechanism responsible for the upregulation of Ca^2+^-permeable AMPARs allowed the recovery of cell-specific AP changes in peripheral inflammation. Selective inhibition of Ca^2+^-permeable AMPARs in the spinal cord alleviated nociceptive hypersensitivity, both thermal and mechanical modalities, in animals with peripheral inflammation. Thus, Ca^2+^-permeable AMPARs contribute to shaping APs in DH neurons and nociceptive hypersensitivity. This may represent a neuropathological mechanism in the DH circuits, leading to aberrant signal transfer to other nociceptive pathways.

## 1. Introduction

Persistent or chronic pain is notoriously known for its reluctant treatment. Unlike acute pain being remedied with analgesics of choice, chronic pain remains hardly manageable—the existing routes to alleviate chronic pain are often concomitant with numerous adverse effects. Chronic pain has been thought to be mediated by central sensitisation of the dorsal horn (DH) of the spinal cord, the area primarily involved in pain processing in the central nervous system. Numerous reports have linked the central sensitisation of the DH to pain of various origins [1,2,3,4,5], considering this phenomenon as a specific form of spinal plasticity [6,7,8,9].

Multiple mechanisms contribute to spinal plasticity encoding nociceptive processing within the DH. Among those remarkable, are the changed expression of glutamate receptors [10,11,12,13], altered activity and subtype composition of voltage-gated channels (for instance, sodium and potassium channel subtypes, calcium and chloride channels) [14,15,16,17,18], reduced glutamate clearance [19], loss of GABAergic inhibition and differential local chloride homeostasis [20,21,22], an unbalanced ratio of synaptic excitation and inhibition [5,23], morphological changes in excitatory synaptic connections [24,25], and others. Pain of different origins can share traits and overlap with some common mechanisms but also have major differences in engaging neuronal circuits and signalling/molecular pathways that underlie hypersensitivity and hyperactivation at the cellular–subcellular level [26,27]. Inflammatory pain is associated with numerous pathologies; however, surprisingly, little is known about the neuropathological mechanisms mediating inflammatory pain compared, for instance, to much deeper investigated neuropathic pain.

Peripheral inflammation triggered by locally released inflammatory mediators and signalling molecules causes an increased firing of primary nociceptive afferents [28]—peripheral sensitization [7,29]—those transfer action potential (AP) patterns to the DH of the spinal cord. There was reported an increased level of pro-inflammatory mediators within the spinal cord [30,31] and changes in some of the intrinsic properties of the superficial (lamina I-II) DH neurons, such as decreased input resistance [32], augmented spontaneous activity [5], and synchronous burst firing [33]. The lamina I-II DH neurons are in their majority interneurons, which make the first-order synapses with the nociceptive afferents and are primarily involved in computing incoming sensory inputs before conveying the output signals to the higher central pathways. We have established earlier that peripheral inflammation changes excitatory neurotransmission in the DH; moreover, it occurs in a cell-specific manner [5]. It has emerged that cell-dependent changes in intrinsic excitability represent a common mechanism of central sensitisation in various animal models [32,34,35,36].

Our studies of the mechanistic basis of inflammatory pain [5,11,37] have revealed changes in the AMPA receptor (AMPAR) functioning in lamina I-II DH neurons. This class of glutamate receptors is of particular interest due to the AMPAR’s key role in synaptic excitability and plasticity, especially given the notable contribution of Ca^2+^-permeable AMPARs to inflammatory pain maintenance [8]. The molecular mechanism of changed AMPAR functioning includes protein kinase C alpha (PKCα)-dependent phosphorylation of the GluA2 subunit that disrupts binding of the receptor to its synaptic anchoring protein. Eventually, GluA2-containing (Ca^2+^-impermeable) AMPARs internalise from nociceptive synapses [12], and Ca^2+^-permeable AMPARs are upregulated, as reported by different groups [11,13,38,39,40,41,42]. Our strategies to target this mechanism via pharmacological inhibition of spinal PKCα [39] or gene-silencing [37,39] alleviated nociceptive hypersensitivity in rats with persistent peripheral inflammation. On the cellular level, it was accompanied by remedied AMPAR-mediated currents in lamina I-II DH neurons. Despite the progress, it remains, however, unknown what the possible contribution upregulated Ca^2+^-permeable AMPARs have to the changed neuronal excitability, i.e., neuronal firing activity in the superficial DH in inflammatory pain. Furthermore, it is important to understand whether cell-specific excitability that shapes nociceptive computation within the DH relates to Ca^2+^-permeable AMPARs which are upregulated in lamina I-II DH neurons in inflammatory pain. To fill these gaps, we performed whole-cell electrophysiology and assessed the parameters of APs generated by different classes of DH neurons in control and inflammatory pain in a model of long lasting peripheral inflammation induced by complete Freund’s adjuvant (CFA). To dissect a possible role of Ca^2+^-permeable AMPARs, we utilised a genetic approach to knock down the molecular mechanism of Ca^2+^-permeable AMPAR upregulation in DH neurons in rats with persistent CFA-induced peripheral inflammation. Finally, we assessed the therapeutic effects of selective inhibition of Ca^2+^-permeable AMPARs in the spinal cord on inflammatory pain of different modalities.

## 2. Results

### 2.1. Peripheral Inflammation Causes Neuronal Hyperexcitability in the Superficial DH

**Figure 1:**
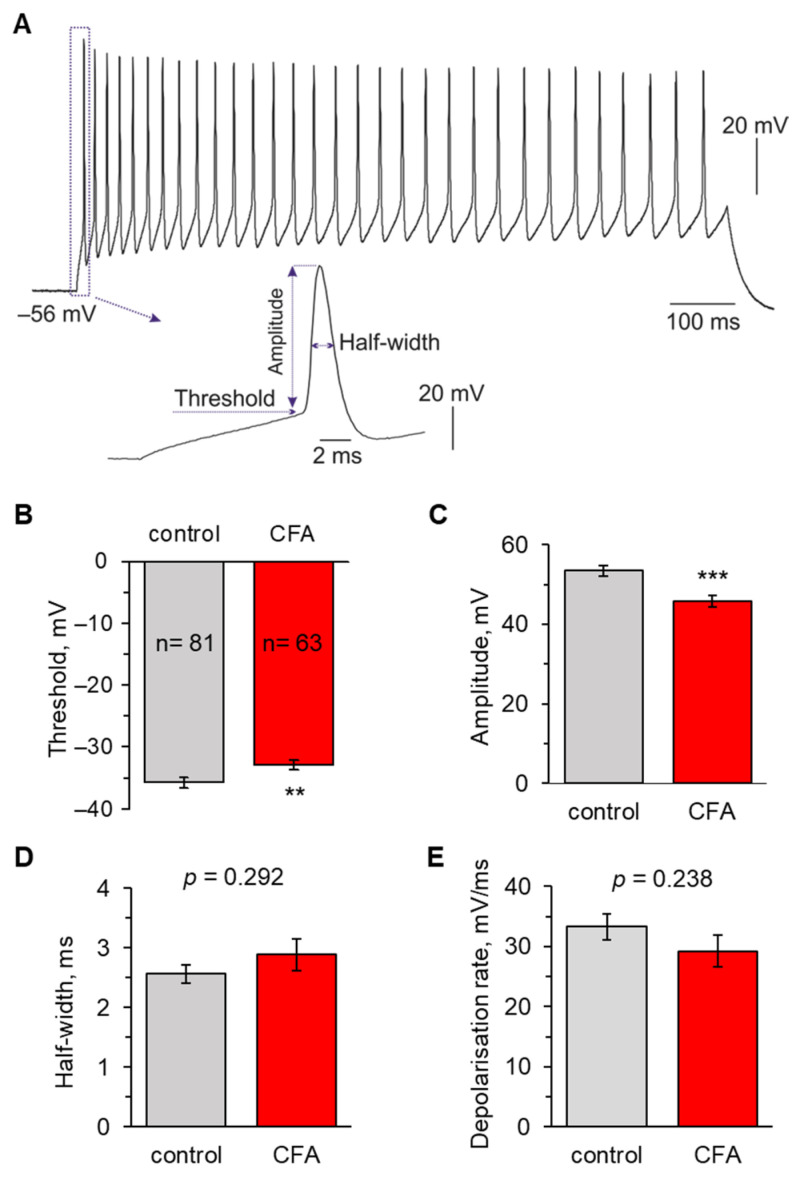
Peripheral inflammation increases the DH neuronal excitability. (**A**) An example of a current-clamp recording of neuronal firing (top) and analysis of the first action potential (AP) in the pulse train (bottom). (**B**) Statistical summary revealed a depolarising shift in the threshold of APs in DH neurons at 1 d post-CFA. (**C**) Analysis of APs showed a decreased amplitude in DH neurons at 1 d post-CFA. (**D**,**E**) Statistical summary of the kinetics of APs generated by DH neurons showed no significant differences in the half-width (**D**) or the depolarisation rate (**E**) between the control and inflamed groups. All data are mean with SEM; the number of recorded cells per group is indicated. ** *p* < 0.01, *** *p* < 0.001 (unpaired two-tailed *t*-test).

We first assessed the excitability of lamina I-II DH neurons by recording evoked APs in current mode to evaluate the parameters of individual APs in DH neurons from control animals and rats with inflammatory-induced peripheral hypersensitivity. Whole-cell recordings were made from lamina I-II neurons in the DH ipsilateral to the plantar injection of saline (control group) or CFA (peripheral inflammation) on day one after injection, a time-point representing persistent nociceptive hypersensitivity [37,43,44]. To elicit firing, we injected a depolarising current of stepwise increasing intensity into a tested neuron; the parameters of individual APs were analysed for the first AP generated in the train (Figure 1A).

We found that peripheral inflammation caused a significant shift in the AP threshold in lamina I-II neurons. The threshold was −35.7 ± 0.8 mV (n = 81) in control versus −32.9 ± 0.7 mV (n = 63) in the CFA-inflamed group (*p* < 0.01; Figure 1B). The depolarising shift in ~3 mV indicates that peripheral inflammation increases the DH neuronal excitability. The AP properties were measured on rheobase (minimum stimulus required to trigger AP generation), which also showed a tendency to decrease in the CFA group (17 ± 4 pA in the control versus 10 ± 1 pA in 1 day post-CFA, *p* = 0.194 Mann–Whitney *U*-test). This, together with a depolarising shift in the threshold, would increase an overall level of neuronal excitability in the superficial DH.

The AP amplitude decreased in lamina I-II neurons in peripheral inflammation: from 53.4 ± 1.4 mV in the control to 45.8 ± 1.4 mV at 1 d post-CFA (*p* < 0.001; Figure 1C). At the same time, there were no significant differences in the AP kinetics between the experimental groups. The depolarising rate was 33.3 ± 2.1 ms/mV (n = 81) in the control and 29.2 ± 2.7 ms/mV (n = 63) in the CFA-inflamed animals (*p* = 0.238); the AP half-width was 2.6 ± 0.2 ms and 2.9 ± 0.3 ms (*p* = 0.292), respectively (Figure 1D,E). Similar kinetics suggests that major ion-channel-mediated conductances that shape APs are not altered in the lamina I-II neurons after peripheral inflammation.

### 2.2. Peripheral Inflammation Specifically Alters Excitability and AP Parameters in the Lamina I-II DH Neurons

**Figure 2:**
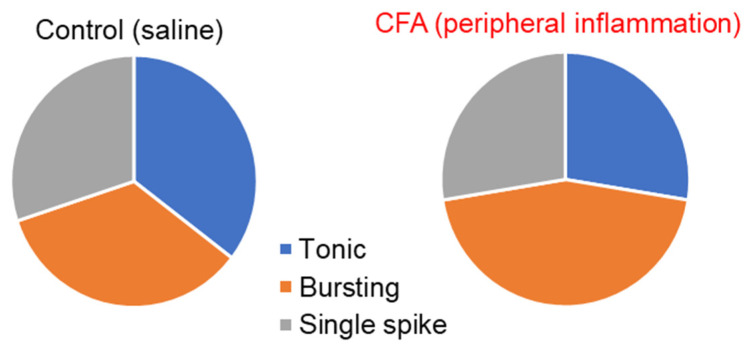
The distribution of neuronal subtypes based on the firing pattern recorded in the superficial DH of the spinal cord in the control (healthy) animals (left) and rats with persistent peripheral inflammation (1-day post-CFA, right). The proportions between the three neuronal classes recorded in the superficial DH did not significantly differ between the control (**left**) and CFA-inflamed (**right**) groups of animals (*p* = 0.309, chi−square test).

Using previously established criteria [5,11,37,45,46], we next classified the recorded DH neurons by firing patterns as tonic firing, initial bursting (or adaptive firing), and single spiking. Among the lamina I-II DH neurons recorded in physiological conditions, tonic firing and initial bursting patterns dominated (tonic firing: 35.4% (40/113); initial bursting: 34.5% (39/113); single spiking: 30.1% (34/113). The CFA-induced peripheral inflammation did not significantly change the proportion between the neuronal classes (*p* = 0.309, chi-square test), although there was a tendency to the more dominant initial bursting responses (44.8% (39/87) at 1 d post-CFA versus 34.5% (39/113) in the control, *p* = 0.344, chi-square test), and a decrease in tonic firing (27.6% (24/87) versus 35.4% (40/113), respectively, *p* = 0.374, chi-square test) in peripheral pain hypersensitivity (Figure 2).

**Figure 3:**
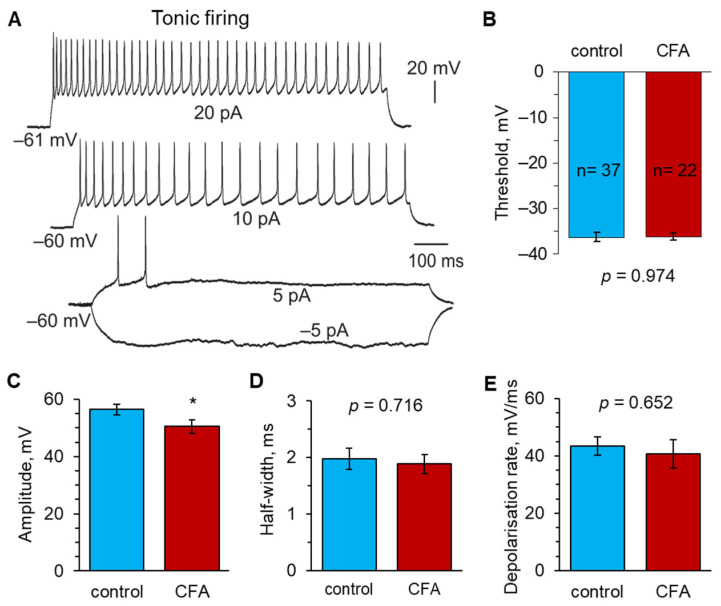
Peripheral inflammation does not increase the excitability of lamina I−II neurons exhibiting tonic firing. (**A**) An example of current-clamp recordings made from a superficial DH neuron with a tonic firing pattern of discharge in response to a stepwise increase in depolarising currents of the indicated intensity. (**B**) Statistical summary of the AP threshold in tonic firing neurons in control animals and those with peripheral inflammation at 1 d post-CFA. (**C**) Peripheral inflammation decreases the spike amplitude in tonic firing lamina I−II neurons. (**D**,**E**) Statistical summary of the kinetics of APs generated by tonic firing DH neurons for the half-width (**D**) and the depolarisation rate (**E**) in the control and CFA-inflamed groups. All data are means with SEM; the number of recorded cells is indicated. * *p* < 0.05 (unpaired two-tailed *t*-test).

The parameters of individual APs were analysed for each neuron across the defined classes. In tonic firing neurons (Figure 3A), peripheral inflammation did not change the AP threshold, which had the same value as in the control, non-inflamed animals (−36.3 ± 1.0 mV, n = 37 in the control versus −36.2 ± 0.8 mV, n = 22 at 1 d post-CFA, *p* = 0.974; Figure 3B). The spike kinetics were also unchanged between the groups of animals (the half-width: 2.0 ± 0.2 ms in the control versus 1.9 ± 0.3 ms at 1 d post-CFA, *p* = 0.716, Figure 3D; the depolarising rate: 43.4 ± 3.2 ms/mV versus 40.7 ± 5.0 ms/mV, *p* = 0.652, Figure 3E; respectively). However, there was a significant drop in the AP amplitude in peripheral inflammation (56.4 ± 1.8 mV, n = 37 in the control versus 50.5 ± 2.3 mV, n = 22 at 1 d post-CFA, *p* < 0.05; Figure 3C).

**Figure 4:**
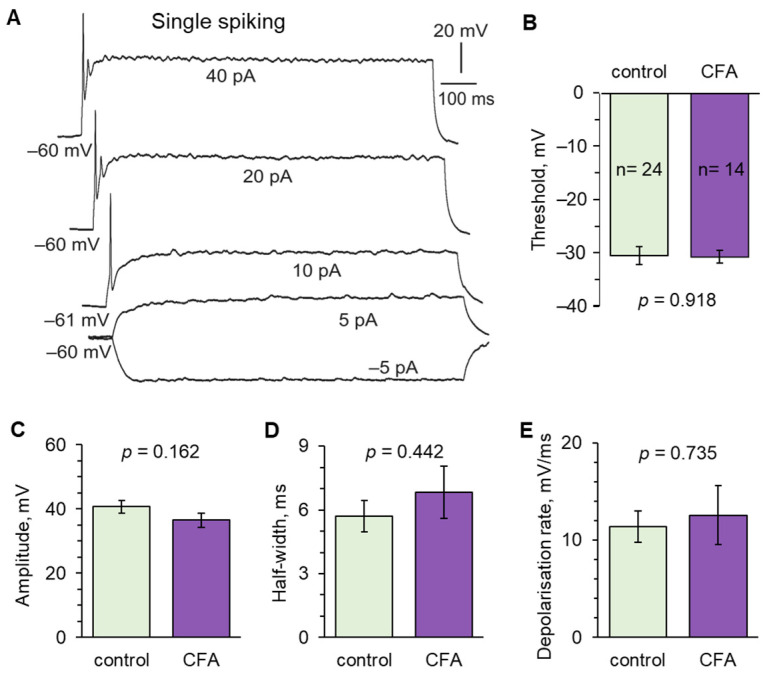
Peripheral inflammation does not affect firing by single spiking lamina I−II DH neurons. (**A**) An example of current-clamp recordings from a DH neuron displaying a single spiking pattern of discharge in response to a stepwise increase in depolarising currents of the indicated intensity. (**B**) Statistical summary of the AP threshold in single spiking DH neurons in the control and 1 d post-CFA groups. (**C**) Summary of the spike amplitude in the control and peripheral inflammation. (**D**,**E**) Statistical summary of the kinetics of APs analysed for the half-width (**D**) and the depolarisation rate (**E**) in the control and inflamed groups. All data are means with SEM; the number of recorded cells is indicated. Unpaired two-tailed *t*-test is indicated).

In single spiking neurons (Figure 4A), there were no differences in the AP parameters, for the spike threshold (Figure 4B) nor kinetics (Figure 4D,E), between the control (n = 24) and inflamed cohorts (n = 14). The spike amplitude also did not significantly change in persistent peripheral inflammation (40.7 ± 2.0 mV in the control versus 36.5 ± 2.1 mV at 1 d post-CFA, *p* = 0.162; Figure 4C).

**Figure 5:**
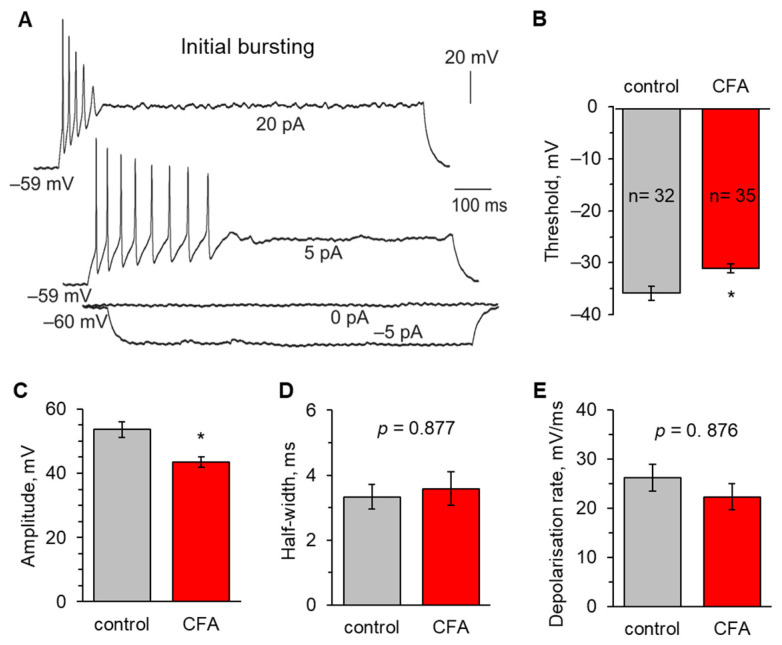
Peripheral inflammation increases the excitability of initial bursting lamina I−II DH neurons. (**A**) An example of current-clamp recordings from a neuron displaying an initial bursting firing pattern in response to a stepwise increase in depolarising currents of the indicated intensity. (**B**) Peripheral inflammation shifts the AP threshold to more depolarised values. (**C**) Peripheral inflammation decreases the AP amplitude in initial bursting neurons. (**D**,**E**) Statistical summary of the kinetics of APs analysed for the half-width (**D**) and the depolarisation rate (**E**) in the control and inflamed groups. All data are means with SEM; the number of recorded cells is indicated. * *p* < 0.05 (unpaired two-tailed *t*-test).

In contrast to the above, initial bursting neurons (Figure 5A) displayed marked changes in both AP threshold and amplitude in persistent peripheral hypersensitivity. The threshold was significantly lowered in initial bursting neurons at 1 d post-CFA (−35.7 ± 1.4 mV, n = 32 in the control versus −31.0 ± 0.9 mV, n = 35 at 1 d post-CFA, *p* < 0.05; Figure 5B). Similarly, there was a drop in the AP amplitude in inflammatory pain conditions (53.6 ± 2.3 mV in the control versus 43.5 ± 1.7 mV at 1 d post-CFA, *p* < 0.05; Figure 5C). A depolarising shift in ~4.7 mV indicated an increased excitability that this class of DH neurons exhibited in response to nociceptive drive from the periphery. Nevertheless, there were no changes in the kinetics of APs generated by these neurons in the control or peripheral inflammatory conditions. The AP half-width was 3.3 ± 0.4 ms in the control versus 3.6 ± 0.5 ms at 1 d post-CFA (*p* = 0.877; Figure 5D), and the depolarising rate was 26.2 ± 2.8 ms/mV versus 22.3 ± 2.7 ms/mV (*p* = 0.876; Figure 5E), respectively.

These findings indicate that persistent peripheral inflammation drove cell-specific changes in the firing activity of superficial DH neurons in response to increased nociceptive inputs from the periphery. Summarising, the nociceptive effects on the superficial DH result in increased excitability of initial bursting neurons but not tonic firing or single spiking neurons. At the same time, no changes in the AP kinetics detected in all the neuronal classes suggest that peripheral inflammation does not deteriorate neuronal capability to maintain firing discharge in any of the classes tested.

### 2.3. Knockdown of the Inflammatory-Induced Upregulation of Ca^2+^-Permeable AMPARs in the Spinal Cord Restores the AP Parameters in Lamina I-II Neurons

**Figure 6:**
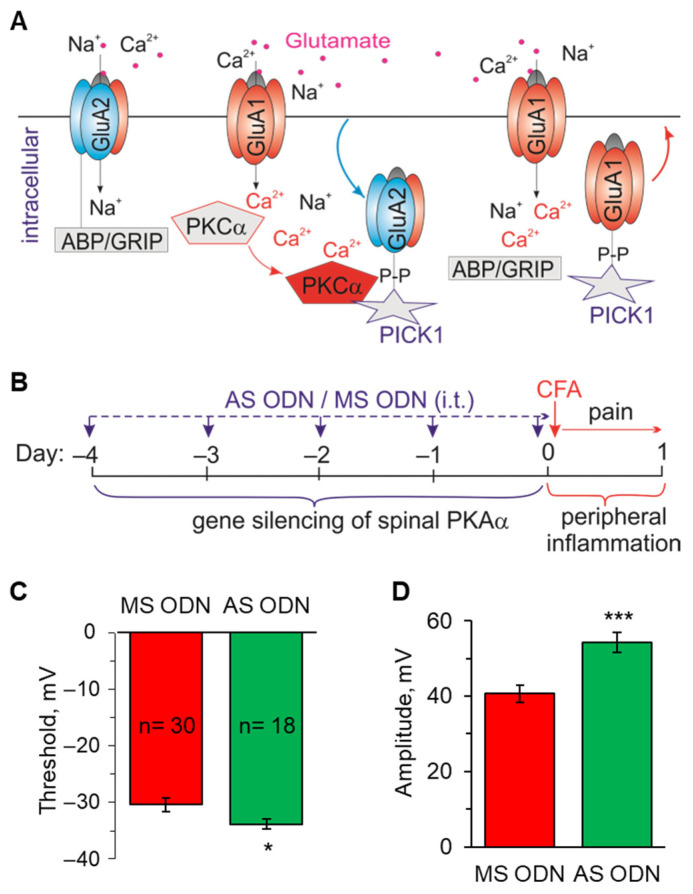
Knockdown of the molecular mechanism mediating the upregulation of Ca^2+^-permeable AMPARs restores the AP parameters in lamina I−II DH neurons. (**A**) A schematic illustration of the cascade of signalling events mediating the internalisation of GluA2-containing, Ca^2+^-impermeable AMPARs from synapses and the upregulation of GluA1-containing, Ca^2+^-permeable AMPARs in lamina I−II neurons in persistent inflammatory pain. ABP/GRIP protein binds to and anchors GluA2 at the membrane; an increase in nociceptive drive from the periphery activates postsynaptic glutamate receptors, increases Ca^2+^ influx into the superficial DH neurons activating PKCα; the latter phosphorylates GluA2 and disrupts the receptor’s binding to ABP/GRIP, which leads to the internalisation of GluA2-containing AMPARs and insertion of GluA1-containing, Ca^2+^-permeable AMPARs in the plasma membrane. (**B**) An experimental scheme of the knocking down of spinal PKCα with AS ODN administered intrathecally (i.t., 10 μL/rat) before the induction of peripheral inflammation daily for 4 days. (**C**,**D**) Statistical summary of the AP threshold (**C**) and the amplitude (**D**) in lamina I−II DH neurons in the CFA-inflamed animals after AS− or MS−ODN treatment. All data are means with SEM; the number of recorded cells is indicated. * *p* < 0.05, *** *p* < 0.001 (unpaired two-tailed *t*-test).

Persistent peripheral inflammation upregulates Ca^2+^-permeable AMPARs at the synapses between lamina I-II neurons and nociceptive fibres and in the extrasynaptic membranes [11,12,13]. To assess the contribution of Ca^2+^-permeable AMPARs to changed AP parameters, we implemented the experimental design of knocking down the molecular cascade responsible for the receptors’ surface expression (Figure 6A), as described in detail in our previous studies [37,39]. Gene-silencing was performed in vivo via spinal administration of AS ODN (experimental group) or MS ODN (control group) for a few consecutive days prior to the induction of peripheral inflammation (Figure 6B). This treatment scheme effectively restored the AMPAR-mediated currents in lamina I-II DH neurons together with nociceptive hypersensitivity in rats with peripheral inflammation reported in our previous works [37,39]. Here, we recorded the APs generated by lamina I-II DH neurons at day one post-CFA to match the time-point of inflammatory pain maintenance as above.

The knockdown approach recovered the impaired AP parameters in the superficial DH neurons. In grouped neurons, the AP threshold was −33.9 ± 0.9 mV (n = 18) in the cohort of CFA-inflamed animals treated with AS ODN, whereas the threshold was −30.4 ± 1.2 mV (n = 30) in the CFA-inflamed animals treated with MS ODN (*p* < 0.05; Figure 6C). These results demonstrate the effect of targeting spinal Ca^2+^-permeable AMPARs on neuronal hyperexcitability in the superficial DH in conditions of peripheral inflammation. Further to the alleviated excitability, the amplitude of APs at 1 d post-CFA recovered after AS ODN treatment compared with that after MS ODN (54.2 ± 2.6 mV versus 40.7 ± 2.3 mV, *p* < 0.001, respectively; Figure 6D). The spike amplitude in superficial DH neurons from the CFA-inflamed animals treated with AS ODN was comparable to that in the control, non-inflamed group of animals (53.4 ± 1.4 mV; Figure 1C).

Thus, Ca^2+^-permeable AMPARs markedly contribute to the inflammatory-driven neuronal hyperexcitability in the superficial DH by shifting the threshold of firing in lamina I-II neurons to more depolarised potentials in conditions of persistent peripheral inflammation.

### 2.4. Knockdown of Upregulated Ca^2+^-Permeable AMPARs in the Spinal Cord Restores the AP Parameters in Lamina I-II Neuronal Classes in Persistent Peripheral Inflammation

We next examined whether Ca^2+^-permeable AMPARs play a role in the cell-specific changes of AP parameters in lamina I-II DH neurons. Here, we focused on the tonic firing and initial bursting neuronal classes, in which changes were found as described above.

**Figure 7:**
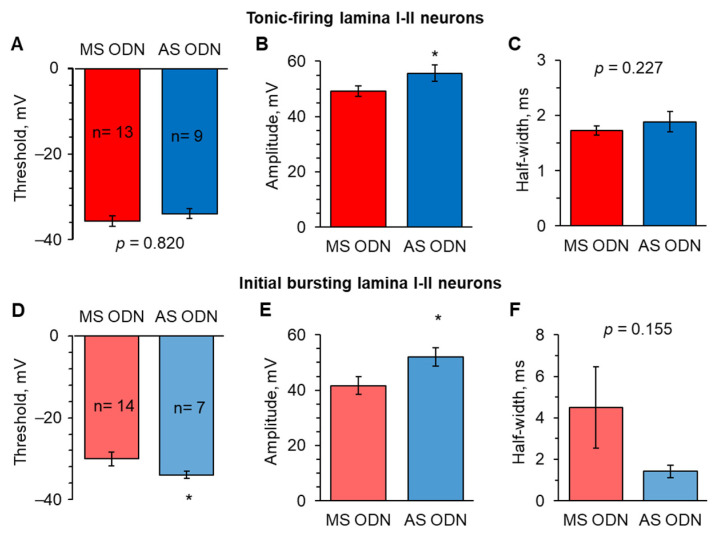
Knockdown of the molecular mechanism mediating the inflammatory-induced upregulation of Ca^2+^-permeable AMPARs restores cell-specific changes in the AP parameters in lamina I−II neurons. (**A**) Statistical summary of the AP threshold in tonic firing lamina I−II neurons in the CFA−inflamed animals after AS− or MS−ODN treatment. (**B**,**C**) Summary of the AP amplitude (**B**) and the half−width (**C**) in tonic firing neurons in different experimental groups. (**D**) AS ODN treatment recovered the AP threshold in initial bursting lamina I−II neurons in peripheral inflammation. (**E**,**F**) Same as in B and C, but for the initial bursting lamina I−II neurons. All data are means with SEM; the number of recorded cells is indicated. * *p* < 0.05 (unpaired two-tailed *t*-test).

In tonic firing neurons, AS ODN treatment recovered the AP amplitude at 1 d post-CFA compared to that in the CFA-inflamed animals treated with MS ODN (55.7 ± 2.9 mV, n = 13 after AS ODN versus 49.2 ± 2.0 mV, n = 9 after MS ODN, *p* < 0.05; Figure 7B). The magnitude of APs in these neurons recovered back to levels observed in the control, non-inflamed group (56.4 ± 1.8 mV; Figure 3C). At the same time, there were no significant changes in the AP threshold between the CFA-inflamed animals treated with AS ODN or MS ODN (−34.1 ± 1.2 mV versus −35.8 ± 1.2 mV, respectively, *p* = 0.820; Figure 7A) and kinetics (half-width: 1.9 ± 0.2 ms versus 1.7 ± 0.1 ms, respectively, *p* = 0.227; Figure 7C). The latter is in full agreement with no effect of peripheral inflammation on these parameters in tonic firing neurons described above.

In the initial bursting neurons, AS ODN treatment (knockdown of upregulated Ca^2+^-permeable AMPARs) fully recovered the two parameters of APs, which became changed by peripheral inflammation: the threshold and the amplitude. At 1 d post-CFA, the AP threshold was significantly lower—more hyperpolarised—after AS ODN treatment than MS ODN (−34.0 ± 0.8 mV, n = 7 versus −30.1 ± 1.7 mV, n = 14, respectively, *p* < 0.05; Figure 7D). So were the changes in the AP amplitude (52.1 ± 3.3 mV after AS ODN versus 41.6 ± 3.2 mV after MS ODN, *p* < 0.05, respectively; Figure 7E). In initial bursting neurons from the CFA-inflamed animals treated with AS ODN, the AP amplitude had values similar to that in the non-inflamed group (53.6 ± 2.3 mV; Figure 5C).

### 2.5. Selective Inhibition of Spinal Ca^2+^-Permeable AMPARs Alleviates Inflammatory Pain of Thermal Modality

**Figure 8:**
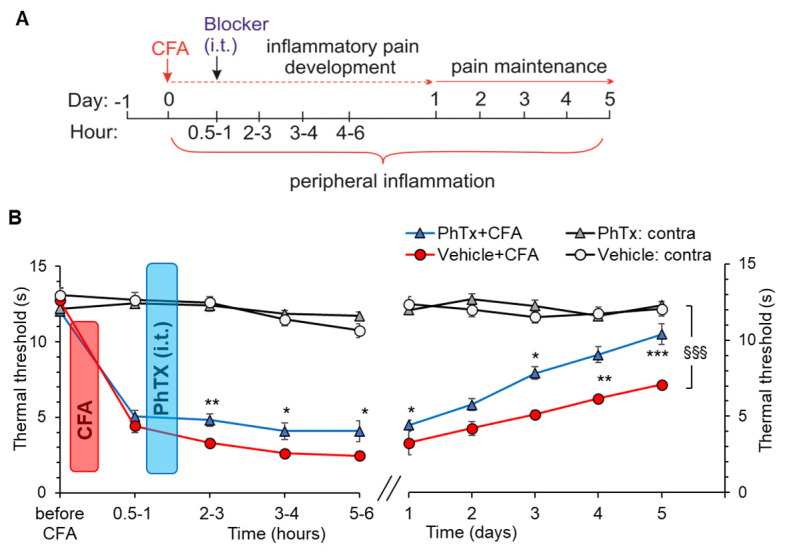
Selective inhibition of Ca^2+^-permeable AMPARs in the spinal cord alleviates inflammatory pain of thermal modality in rats with peripheral inflammation. (**A**) An experimental scheme of post-treatment with philantotoxin−74 (PhTx, 1 μM), given intrathecally (i.t.) after the development of peripheral hypersensitivity in rats induced by the intraplantar injection of CFA. (**B**) The time course of changes in the paw withdrawal latency in response to heat stimulus (thermal nociceptive threshold) before and following i.t. administration of the blocker in the experimental groups of animals with ipsilateral CFA-induced peripheral inflammation. The time points tested and the scheme of treatment are as shown on A. * *p* < 0.05, ** *p* < 0.01, *** *p* < 0.001 versus the corresponding time in the CFA-inflamed group without PhTx; ^§§§^ *p* < 0.001 versus to the corresponding time on the contralateral (non-inflamed) side.

To selectively inhibit Ca^2+^-permeable AMPARs in the spinal cord, philantotoxin-74, a highly potent antagonist of these receptors, was administered into the lumbar spinal cord after the confirmation of developing peripheral nociceptive hypersensitivity in rats (Figure 8A). We determined the therapeutic effect of philantotoxin on inflammatory pain development (within several hours post-CFA) and maintenance (up to 5 days after the induction of inflammatory hypersensitivity). Consistent with our previous reports [37,39,43,44], intraplantar injection of CFA produced a sharp drop in the thermal nociceptive threshold on the ipsilateral to injection hind paw within 30 min after the injection in each animal tested (n = 9; Figure 8B). The hypersensitivity further developed until reaching a plateau by day one post-CFA. This was maintained over several days until it slowly recovered [37,39,43,44].

Philanthotoxin administered shortly after the confirmation of developing nociceptive hypersensitivity (30 min post-CFA) significantly alleviated the thermal nociceptive threshold in rats. The antinociceptive effect of 1 μM philanthotoxin appeared fast—the thermal threshold of the inflamed paw increased within 30–60 min after drug administration by ~46% (*p* = 0.01, *n* = 7) compared with CFA-inflamed animals without treatment (Figure 8B). The effect was continuous; there was a tendency to its time-dependent increase. In particular, the threshold increased by ~55% (*p <* 0.05) after 3–4 h and by ~66% (*p =* 0.05) after 5–6 h post-CFA. The antinociceptive effect remained similar after day 1 post-CFA (*p <* 0.05) and over the following days of testing. At days 4 and 5 post-CFA, the thermal nociceptive threshold recovered to 9.0 ± 0.6 s and 10.4 ± 0.17 s (n = 7), respectively, reaching the control (pre-inflammatory) levels in animals that received a single injection of the blocker. In contrast, the threshold was only 6.2 ± 0.34 s and 7.1 ± 0.62 s, in CFA-inflamed animals without philanthotoxin, respectively.

There were no significant differences in the thermal nociceptive threshold on the contralateral hind paw over the entire duration of testing in any group of animals (Figure 8B). The latter indicates no adverse effects of philanthotoxin on basal thermal sensitivity produced by the inhibition of Ca^2+^-permeable AMPARs in the spinal cord.

Thus, selective inhibition of Ca^2+^-permeable AMPARs in the spinal cord alleviates the inflammatory-induced nociceptive hypersensitivity of thermal modality. This further confirms the role of Ca^2+^-permeable AMPARs in DH hyperexcitability under peripheral inflammation.

### 2.6. Inhibition of Ca^2+^-Permeable AMPARs in the Spinal Cord Alleviates Inflammatory Pain of Mechanical Modality

**Figure 9:**
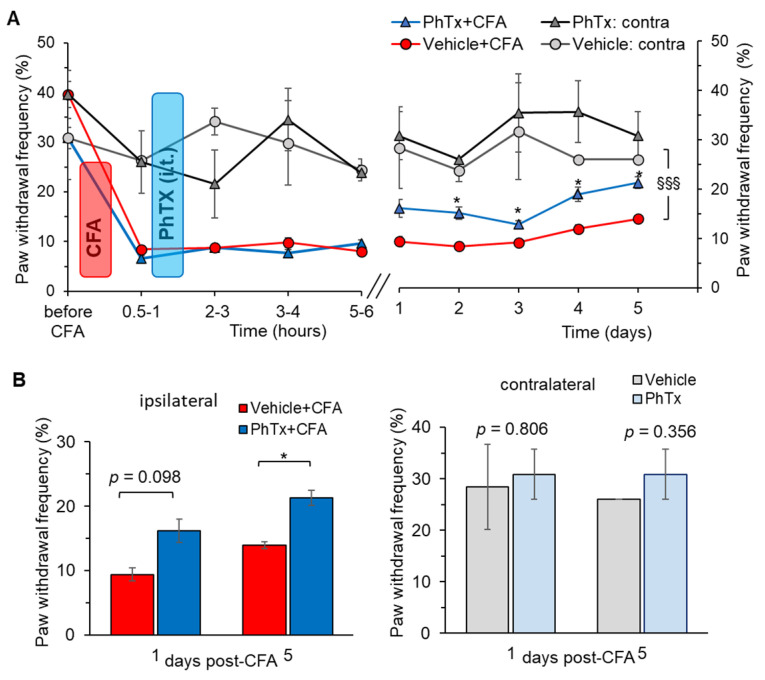
Selective inhibition of Ca^2+^-permeable AMPARs in the spinal cord alleviates inflammatory pain of mechanical modality in rats with peripheral inflammation. (**A**) The time course of changes in the paw withdrawal frequency—the mechanical nociceptive threshold—following inhibition of Ca^2+^-permeable AMPARs with philantotoxin−74 (PhTx, 1 μM), given intrathecally (i.t.) after the development of inflammatory-induced peripheral mechanical hypersensitivity. The experimental scheme was as shown in Figure 8A. The withdrawal frequency normalised to maximum response (100%). * *p* < 0.05 compared to the corresponding time in the CFA-inflamed group without PhTx; ^§§§^ *p* < 0.001 compared to the corresponding time on the contralateral (non-inflamed) side. (**B**) Summary of the paw withdrawal frequency on days 1 and 5 after CFA. * *p* < 0.05 versus as indicated.

Finally, we determined the therapeutic effect of philanthotoxin on inflammatory pain of mechanical modality using the method of von Frey monofilaments. Mechanical hypersensitivity was assessed within several hours after the philanthotoxin injection (inflammatory pain development) and then daily up to 5 days post-CFA (pain maintenance). Consistent with our previous reports [37,43], injection of CFA produced robust allodynia and mechanical hypersensitivity in response to stimulation with von Frey monofilaments on the ipsilateral but not on the contralateral side (Figure 9A). Philanthotoxin (1 μM) alleviated the mechanical nociceptive hypersensitivity in animals with peripheral inflammation on day 1, although the effect did not reach statistical difference: the nociceptive threshold was 16.2 ± 1.8% after philanthotoxin versus 9.4 ± 1.1% after vehicle (*p* = 0.098; Figure 9B). However, the antinociceptive effect of philanthotoxin was significant from day 2 (the threshold was 15.1 ± 1.3%, n = 7 versus 8.4 ± 0.5%, n = 5, *p* < 0.05, respectively).

Pain relief remained stable over the next tested days (*p* < 0.05; Figure 9A). Spinal administration of philanthotoxin resulted in the recovery of the mechanical threshold in the CFA-inflamed animals by day 5, so the mechanical sensitivity of the ipsilateral, inflamed paw was similar to that of the contralateral, non-inflamed paw (*p* > 0.05; Figure 9A,B). These data demonstrate that selective inhibition of Ca^2+^-permeable AMPARs with philanthotoxin significantly shortened mechanical pain maintenance.

Importantly, animals did not display any difference with regard to their basal mechanical sensitivity following the spinal administration of philanthotoxin at any time-point tested (the nociceptive threshold measured on the contralateral side). This indicates that inhibition of spinal Ca^2+^-permeable AMPARs with philanthotoxin produced no detectable adverse effects on basal tactile sensation. This is similar to the observation above for basal thermal sensitivity.

## 3. Discussion

Treatment of chronic pain requires mechanism-based therapies to provide an effective remedy for pain, yet not affect normal tactile sensitivity or give rise to adverse effects. Chronic pain is a complex pathology that overlaps multiple mechanisms and signalling pathways which become impaired; therefore, many routes have been actively explored over the last years for an effective interception of pain at both the cellular and systemic levels. This study is a continuation of our previous works on targeting Ca^2+^-permeable AMPARs in the spinal cord to prevent the nociceptive-induced upregulation of these receptors, concomitant with the DH hyperexcitation and inflammatory pain hypersensitivity [8]. Here, we focused on the role of Ca^2+^-permeable AMPARs in changed firing by lamina I-II DH interneurons in peripheral inflammatory-induced nociceptive hypersensitivity, using a combination of contemporary approaches, such as whole-cell electrophysiology, in vivo assessment of nociceptive threshold (both thermal and mechanical modalities), and knockdown (gene-silencing) to prevent the nociceptive-induced upregulation of Ca^2+^-permeable AMPARs in DH neurons.

Our previous studies of the central mechanisms of chronic pain development and maintenance unveiled the vital role of upregulated Ca^2+^-permeable AMPARs in inflammatory pain [5,8,11,37,39]. The mechanism induced by peripheral sensitisation following tissue inflammation is mediated by changes in the dynamic trafficking of AMPARs at the synapses between the lamina I-II DH neurons and nociceptive fibres where the persistent afferent drive from the periphery facilitates the internalisation of postsynaptic GluA2-containing (Ca^2+^-impermeable) AMPARs. The latter causes an increased proportion of Ca^2+^-permeable receptors and an augmented Ca^2+^-influx into lamina I-II DH neurons. We found this mechanism at the synapses and extrasynaptic membranes via the promoted insertion of GluA1-containing, Ca^2+^-permeable AMPARs [47]. Eventually, the AMPAR-mediated Ca^2+^-conductance increases [11,48,49]—it drives neuronal excitability, the DH neurons to become hyperexcitable, and the DH neuronal circuits to hyperactivate [5], causing the DH sensitisation linked to pain chronification [8]. Our current results show that this occurs due to a shift in the AP threshold to more depolarising potentials; also, lamina I-II DH neurons exhibit a lower rheobase in persistent inflammatory hypersensitivity. Altogether it provides new insights into neuronal hyperexcitability in inflammatory pain conditions, albeit confirming the available knowledge of central hyperexcitability by peripheral sensitisation in pain of neuropathic origin (for review, see [6,9]). Our study demonstrates the critical role of upregulated Ca^2+^-permeable AMPARs in neuronal hyperexcitability in persistent inflammatory pain. To delineate the contribution of Ca^2+^-permeable AMPARs to changed AP parameters in superficial DH interneurons, we implemented highly selective genetic inhibition of the signalling cascade responsible for the receptors’ upregulation (i.e., knocking down PKCα, which phosphorylates the receptor subunits, thus facilitating the receptors’ trafficking—GluA2 internalisation / GluA1 insertion). This approach allowed us to confirm that Ca^2+^-permeable AMPARs shape changed neuronal firing activity in the superficial DH in inflammatory pain. Inhibiting nociceptive upregulation of Ca^2+^-permeable AMPARs in the spinal cord prevented changes in the AP parameters in the lamina I-II neurons in animals with peripheral inflammation. This is consistent with our previous findings of restored postsynaptic currents and total AMPAR-mediated currents in lamina I-II neurons in CFA-inflamed animals following the same experimental treatment [37,39].

Other subtypes of glutamate receptors contribute to peripheral and central sensitisation [50]. Many studies have demonstrated that activation of NMDA receptors (NMDARs), a subclass of ionotropic glutamate receptors, mediate changed nociceptive transmission and chronic pain [51], including CFA-induced orofacial pain [52], visceral and post-operative pain [53]. Similar to AMPARs, the NMDAR-dependent mechanism initiates complex intracellular cascades through Ca^2+^-influx and activation of protein kinases [12] (e.g., PKA, PKC, casein kinase II, tyrosine kinases, others (for review see [54,55]) that modulate membrane excitability and neuronal excitation. The PKC-dependent phosphorylation of NR2B at the Ser1303 and Ser1323 enhanced the current and reduced Mg^2+^ blockade of the receptor [56,57], whereas phosphorylation at the Ser896 increased membrane translocation of the NMDAR [58]. The other subtype of ionotropic glutamate receptors—kainate receptors—was also associated with chronic pain [59,60,61].

Engaged neuronal classes and cell-specific excitability shape nociceptive computation within the DH. Although superficial DH interneurons receive inputs from higher brain centres and the periphery, virtually all lamina II interneurons have axons within the spinal cord and are highly interconnected with each other [62,63,64,65] to modulate pain signals within the intrinsic circuitry and transfer the output into the higher brain centres. Thus, the overall level of excitability within the superficial DH, e.g., patterns of firing activity of engaged neuronal classes, would eventually define what nociceptive signal (i.e., facilitated or reduced) will be transmitted in normal sensation or pathological conditions. Likewise, the cell-heterogeneous changes in neuronal excitability would set the threshold to respond to peripheral inputs. In addition, orthodromic propagation of APs generated close to the afferent terminals may enhance the excitability of nociceptors [66]. Our results confirmed the cell-specific changes in AP parameters in peripheral inflammation: the AP threshold was depolarised in the initial bursting neurons, marking these neurons hyperexcitable, whereas no changes in the AP parameters were found in single spiking neurons, but reduced AP magnitude in both classes of tonic firing and initial bursting neurons. The heterogeneous changes in AP parameters and excitability imply that different neuronal classes play differential roles in nociceptive signal processing in inflammatory pain. Identifying the neurotransmitter phenotypes of the recorded neurons, i.e., excitatory or inhibitory interneurons, would be essential to straightforwardly interpret the effects of altered firing activity on nociceptive signal modification in inflammatory pain.

Most of the lamina II interneurons are excitatory, glutamatergic (>70%), and nearly 30% are inhibitory, GABAergic [63,67]. Over the last decades, there have been numerous attempts to identify the specific phenotypes of lamina I-II neurons using various approaches [46,62,67,68,69,70], including advances in purposely designed mouse lines [68,71,72,73]. More recently, specific labelling of parvalbumin-expressing interneurons in PV-Cre knock-in mice has demonstrated that parvalbumin-positive neurons classified as tonic firing or initial bursting neurons were equally split into excitatory and inhibitory phenotypes [74]. Furthermore, the inhibitory phenotype represents a mixed population of GABAergic and glycinergic interneurons in pain-processing circuits, which co-express various neuronal markers [73,75,76]. Therefore, the knowledge of how firing activity pattern refers to the neurotransmitter-related phenotype of superficial DH neurons remains very limited, despite its importance for the whole field. Without this, the neuropathological consequences of the changed AP parameters described above can be as follows. Because peripheral inflammation enhances the excitability of initial bursting neurons, this neuronal class very likely represents mostly the excitatory phenotype—the changed firing activity of these neurons would have pro-nociceptive effects, leading to further sensitisation of the DH circuits in pain conditions. Consistently with this hypothesis, selective ablation of a subpopulation of excitatory DH neurons that express somatostatin [77] or co-express VGLUT3 and Lbx1 [78] resulted in reduced mechanical hypersensitivity induced by nerve injury or inflammation. Assuming that many tonic firing neurons are inhibitory GABAergic interneurons yet overlapping some proportion of excitatory neurons [68,79,80,81], the unchanged AP threshold in these neurons indicates that peripheral inflammation does not produce hyperactivation of inhibitory interneurons. Otherwise, boosted inhibitory control over the intrinsic DH circuits experiencing hyperexcitation would provide the fine-tuning of inhibition of sensory inputs in peripheral hypersensitivity. Likewise, the ablation of a subpopulation of DH inhibitory neurons expressing dynorphin produced spontaneously developed mechanical pain (peripheral allodynia) [77], whereas ablation of inhibitory deep layer DH interneurons expressing the receptor tyrosine kinase enhanced basal and chronic pain [82].

Our current data extend the knowledge of cell-specific nociceptive mechanisms in central pathways at the subcellular/receptor level, demonstrating the role of upregulated Ca^2+^-permeable AMPARs in altered excitability of neuronal classes in the superficial DH. The therapeutic effects of targeting this receptor subtype in the spinal cord include (i) the AP parameters restored in both classes of lamina I-II DH neurons—tonic firing and initial bursting (Figure 7), tentatively inhibitory and excitatory interneurons; (ii) the AMPAR-mediated currents normalised back to a control level, and (iii) inflammatory-induced thermal and mechanical hypersensitivities alleviated in rats with CFA-induced peripheral inflammation [37,39]. Further studies are required to confirm the phenotype of the recorded neurons. Moreover, it is important because specific subpopulations of DH interneurons process itch, thermal or mechanical sensation, or other modalities that may be disrupted differently depending on how the activity changes across cell classes engaged in processing nociceptive inputs within the DH.

Our present (Figure 8 and Figure 9) and previous data on persistent pain in CFA-induced peripheral inflammation [37,43] demonstrated a robust development of hypersensitivity of both thermal and mechanical modalities, which remained persistent for at least several days. Selective inhibition of Ca^2+^-permeable AMPARs in the spinal cord with philanthotoxin, a highly potent antagonist (IC_50_: 263 and 296 nM for homomeric GluA3 and GluA1, respectively), markedly alleviated inflammatory pain. Its effect was assessed for the acute and/or prolonged antinociception, for both thermal and mechanical modalities, by carrying out behavioural tests every hour (5 to 6 h duration) and then daily over the next 5 days post-treatment. Antinociceptive effects were time-dependent, and the inflammatory pain maintenance was shortened for thermal and mechanical hypersensitivities. The capability of antagonists of Ca^2+^-permeable AMPARs to alleviate pain was reported by different studies, including our previous works on the group of dicationic compounds [43]. The antinociceptive effects of the group of organic toxins, to which philanthotoxin belongs with joro spider toxin, argiotoxin, and others, were thoroughly tested for the pre-treatment of injury-evoked allodynia [83,84,85]. Pre-treatment with joro spider toxin or philanthotoxin attenuated the development of secondary mechanical allodynia in a thermal injury model [83,84] and reversed secondary mechanical allodynia in a post-incision pain model [85]. In striking contrast, when administered as a post-treatment, organic toxins did not change the pain maintenance or secondary hyperalgesia in models of post-incision pain [86] or first-degree burns (thermal injury) [87]. Nevertheless, the antinociceptive effect of philanthotoxin was significant in the other animal pain model used as a post-treatment of developing CFA-induced peripheral hypersensitivity. This is coherent with other reports in inflammatory pain, alleviated by antagonists of Ca^2+^-permeable AMPARs [10,88] or the nociceptor-specific deletion of GluA1 [3,89]. Importantly, the selective inhibition of spinal Ca^2+^-permeable AMPARs with philanthotoxin produced no detectable effects on normal tactile sensation in animals in our study, similar to what was reported by others using different antagonists of these receptors [43,88,89].

To summarise, nociceptive-induced upregulation of Ca^2+^-permeable AMPARs in superficial DH interneurons is causally linked to the altered firing activity of cell classes in the DH in inflammatory pain. Targeting the signalling mechanism responsible for the upregulation of these receptors in inflammatory pain allowed to restore the AP parameters in the lamina I-II interneurons—shifting the threshold from more depolarised potentials to its control level in initial bursting neurons and recovering the reduced spike magnitude in both the initial bursting and tonic firing lamina I-II neurons—thus reversing the DH neuronal hyperexcitability associated with inflammatory-induced nociceptive hypersensitivity. Selective inhibition of spinal Ca^2+^-permeable AMPARs in rats with persistent peripheral inflammation alleviated nociceptive hypersensitivity of both thermal and mechanical modalities at the periphery. Our data support targeting Ca^2+^-permeable AMPARs in the spinal cord as an advanced strategy for inflammatory pain treatment in the central pathways.

## 4. Materials and Methods

### 4.1. Animal Care

The animals were male Wistar rats (~1 month old). All animal procedures were approved by the local Animal Ethics Committee in Bogomoletz Institute of Physiology (Kyiv, Ukraine), following the European Commission Directive (86/609/EEC) and ethical guidelines of the International Association for the Study of Pain. We aimed to minimise animals’ discomfort and reduce the number of animals used.

### 4.2. Induction of Peripheral Inflammation

Unilateral peripheral inflammation was induced by intraplantar injection of complete Freund’s adjuvant (CFA, an oil-saline (1:1) emulsion). CFA was injected subcutaneously at a volume of 50–100 µL into the right hind paw, as described in our previous studies [37,39,43,44]. Saline (0.9%) was used as a control. CFA was purchased from Sigma-Aldrich Company Ltd. (St. Louis, MO, USA and Dorset, UK).

### 4.3. Surgical Intrathecal Catheter Implantation

For local delivery to the spinal cord, a catheter was implanted into the lumbar spinal cord region as described in detail previously [43,90]. Briefly, once animals were mounted in a stereotaxic frame system and under stable anaesthesia (mixture of ketamine and xylazine injected at 70 mg kg^−1^ and 15 mg kg^−1^, respectively), a polyethene tube (PE-10) was inserted into the subarachnoid space at the rostral level of the spinal cord lumbar region through an incision in the atlantooccipital membrane. Animals were closely monitored over several days post-surgery (typically 3–5 days or until complete healing of the surgical incision). The lumbar position of the catheter was confirmed in each animal after the termination of behavioural studies or prior to spinal cord slice preparation.

### 4.4. Delivery to the Spinal Cord

For inhibiting spinal AMPARs, we administered philanthotoxin-74, a potent and selective blocker of Ca^2+^-permeable AMPARs, intrathecally (i.t.). The compound inhibits Ca^2+^-permeable AMPARs at high potency (IC_50_: 263 and 296 nM for homomeric GluA3 and GluA1, respectively, which is a 100-fold greater potency than GluA2-containing AMPARs, heteromeric GluA1/2 and GluA2/3 receptors). The blocker was administered at a concentration of 1 μM as a single i.t. injection (10 μL/rat) using a 25-gauge needle connected to a Hamilton syringe. We administered the blocker after confirming the developing nociceptive hypersensitivity in rats with induced peripheral inflammation at 0.5–1 h post-CFA. Injection of the drug was followed by saline administration to flush the catheter. Compounds were purchased from Tocris (Bio-Techne Ltd., Abingdon, UK).

### 4.5. Measuring the Thermal Nociceptive Threshold—The Hargreaves Plantar Test

Peripheral sensitivity to the thermal (heat) stimulus was evaluated in rats using the Hargreaves technique, as described previously [37,43,44]. Briefly, after an animal habituated to the Plexiglas chamber above a lightbox (Ugo Basile Model 7370 Plantar Test), radiant heat was applied to the middle of the plantar surface of one hind paw. The light beam was automatically turned off when the animal lifted its paw. Trials were repeated 3–5 times with an interval between measurements of 3 to 5 min. The time between the stimulus started and the animal lifted its paw—the withdrawal latency—was measured, representing the thermal nociceptive threshold.

### 4.6. Measuring the Thermal Nociceptive Threshold with von Frey Monofilaments

The paw withdrawal responses to repeated mechanical stimuli were measured using the method of von Frey monofilaments as described previously [37,43]. Briefly, after an animal was habituated to an experimental chamber on an elevated mesh screen (at least 10–15 min prior to starting tests), the von Frey monofilaments of different intensities of the stimulus (Bioseb) were applied to the plantar surface of each hind paw. The trial was repeated 10 times for each hind paw with an interval between filament applications for at least 1 min. The percentage of responses was calculated for each trial and defined as the paw withdrawal frequency.

All behavioural tests were performed in a quiet room by an experimenter carrying out tests in a semi-blind-to-drug treatment manner.

### 4.7. Knockdown of the PKCα-Dependent Upregulation of Ca^2+^-Permeable AMPARs in the Dorsal Spinal Cord

For the disruption of the PKCα-mediated upregulation of Ca^2+^-permeable AMPARs in the dorsal spinal cord, we used a gene-silencing approach, as described in our previous studies [37,39]. Briefly, the antisense (AS) oligodeoxynucleotides (ODN) specific to PKC subtype α were used with the following sequence: 5′-GACATCCCTTTCCCCCTCGG-3′; missense (MS) ODN were: 5′-CGTCCTCAGTCGTCCCTCAC-3′, which we used as a control. Animals received a daily injection of AS ODN or MS ODN (10 μg/10 μL) for 3 days. The reduced expression of PKCα protein at the lumbar enlargement segment following the treatment has been confirmed in previous studies [37,39]. AS OND and MS ODN were purchased from ISIS Pharmaceuticals Inc. (Carlsbad, CA, USA).

### 4.8. Spinal Cord Slice Preparation

Spinal cord slices were prepared as described previously [11,37]. Briefly, the spinal cord was dissected and placed in an ice-cold dissection solution containing (in mM) 250 sucrose, 2 KCl, 1.2 NaH_2_PO_4_, 0.5 CaCl_2_, 7 MgCl_2_, 26 NaHCO_3_, 11 glucose, continuously oxygenated with 95% O_2_ and 5% CO_2_. Transverse slices (350 μm thick) with attached dorsal roots (8–15 mm) were cut with an HA752 vibratome (Campden Instruments, Loughborough, UK). Slices were maintained at room temperature in a physiological Krebs bicarbonate solution that contained (in mM) 125 NaCl, 2.5 KCl, 1.25 NaH_2_PO_4_, 2 CaCl_2_, 1 MgCl_2_, 26 NaHCO_3_, 10 glucose, oxygenated with 95% O_2_ and 5% CO_2_ (pH 7.4).

### 4.9. Electrophysiology

Whole-cell recordings (current mode) were made from lamina I-II DH neurons using a Multipatch 700B amplifier controlled with pClamp 9.2 software (Molecular Devices, San Jose, CA, USA). For the recordings, slices were transferred into the continuously perfused recording chamber on an Olympus BX50WI upright microscope (Olympus, Tokyo, Japan). Neurons were visualised with infrared optics using a ×60.09 water-immersion objective. Patch pipettes had a resistance of 4–5 MΩ when filled with an internal solution that contained (in mM) 133 K-gluconate, 5 NaCl, 0.5 MgCl_2_, 10 HEPES-Na, 2 MgATP, 0.1 GTP-Na, 0.5 EGTA (pH 7.2, osmolarity 290 mOsmol). The membrane resistance was constantly monitored by applying a short hyperpolarising pulse (–5 mV).

The lamina I-II DH neurons were categorised according to their discharge patterns in response to the series of depolarising currents (0.5–1 s duration), as described previously [11,37]. The recorded neurons were highly heterogeneous, displaying sustained (tonic) firing, adapting or initial bursting responses, delayed firing, and single spiking discharge patterns. Based on the high expression of the functional A-type potassium channels, varied discharge patterns except the tonic firing could be considered as the adapting firing phenotype [67,69]. For clarity, we arbitrarily grouped all recorded neurons into the main groups of ‘initial bursting’, ‘tonic firing’, and ‘single spiking’ classes. Tonic firing neurons were cells capable of sustained continuous AP generation during the entire depolarising step and increasing the frequency of discharge with increasing stimulus intensity (Figure 1A and Figure 3A).

### 4.10. Statistical Analysis

All data are presented as means ± SEM, with *n* referring to the number of animals tested, or the number of cells recorded. For behavioural studies, statistical difference was analysed by one-way or two-way analysis of variance (ANOVA) followed by the Bonferroni post hoc test where appropriate. For electrophysiological recordings, Student’s *t*-test (two-tailed unpaired) was used to determine statistically significant differences. A chi-square test was used to examine the statistical differences between two categorical variables. A *p* value of less than 0.05 was considered statistically significant for either test.

## Data Availability

All data generated and/or analysed during this study are included in this article.
